# Effect of acetogenin fraction of *Annona muricata* leaves on antioxidant status and some indices of benign prostatic hyperplasia in rats

**DOI:** 10.1080/13510002.2020.1804711

**Published:** 2020-09-02

**Authors:** Patience N. Ogbu, Evelyn O. Ugota, Rita U. Onwuka, Ikechukwu M. Ogbu, Chinyere Aloke

**Affiliations:** a Department of Medical Biochemistry, Faculty of Basic Medical Sciences, Alex Ekwueme Federal University Ndufu-Alike, Ikwo, Nigeria; b Department of Chemistry/Biochemistry, Faculty of Sciences, Alex Ekwueme Federal University Ndufu-Alike, Ikwo, Nigeria

**Keywords:** Benign prostatic hyperplasia, *Annona muricata*, acetogenin fraction, leaves extract, antioxidants

## Abstract

**Objectives:** This work investigated the effect of acetogenin-rich fraction of *Annona muricata* leaves (AFAL) on antioxidant status and some markers of benign prostatic hyperplasia (BPH) in rats.

**Methods:** BPH was experimentally induced in the rats by subcutaneous injection of testosterone propionate (TP, 3 mg/kg) for 28 consecutive days. The rats were administered orally different doses of AFAL (100 and 200 mg/kg) for 7 days. Prostate-specific antigen (PSA), prostate weight, relative prostate weight, prostate protein content and oxidative stress indices of the rats were evaluated.

**Results:** It was observed that 200 mg/kg AFAL significantly reduced the PSA level, mean prostate weights and mean relative prostate weights of the test rats compared to the TP group, and the values were not significantly different from the normal control and group treated with a standard drug. The plant extract also significantly enhanced the antioxidant capacity of the test rats which were evidently compromised in the group that received the exogenous hormone alone. Histopathology of the prostate showed a marked recovery for the test rats after treatment with AFAL.

**Conclusion:** Oral administration of acetogenin-rich fraction of *Annona muricata* leaves ameliorated TP-induced BPH in rats and significantly enhanced the antioxidant capacity of the rats.

## 1. Introduction

Benign prostatic hyperplasia (BPH) is an age-related enlargement of the prostate gland due to the unregulated growth of prostate cells [1,2]. The abnormal proliferation of prostatic stromal cells leads to the formation of discrete nodules in the periurethral region that brings about acute and chronic urinary retention, bladder outlet obstruction, urinary tract infection, urosepsis, bladder stones and hematuria [1,3]. BPH is a progressive disease and its prevalence increases with age.

Several studies have strongly linked etiology of BPH to an imbalance in steroid hormone metabolism, remodeling in ageing prostate, systemic inflammation and oxidative stress associated with metabolic syndrome, among other factors [4,5]. The existing treatment options for BPH include medical therapy with α-blockers or 5α-reductase inhibitors, surgery and phytotherapy [2,6]. While α-blocker relaxes smooth muscles of the prostate and the bladder neck to relieve urinary obstruction caused by an enlarged prostate, 5α-reductase inhibitors prevent the conversion of testosterone to dihydrotestosterone (DHT), thereby leading to the shrinkage of prostate. DHT is an active metabolic product from the reduction of testosterone by 5α-reductase. It plays a critical role in the growth of prostate by binding to the nuclear androgen receptor, thereby inducing synthesis of growth factors that act on prostatic epithelia and stroma, resulting in prostate enlargement [7]. Consequently, inhibitors of 5α-reductase that block the production of DHT ultimately slow down the development of BPH. Common inhibitors of 5α-reductase are pharmacological agents such as dutasteride and finasteride. However, there is strong evidence that some phytochemical agents are also effective inhibitors of 5α-reductase [8] and could contribute significantly to BPH treatment.

Lots of side effects, such as decreased libido, erectile dysfunction, dizziness, retrograde ejaculation and orthostatic hypotension associated with the existing BPH drugs [9,10], have increased interest and research activities on alternative treatment options. The use of phytotherapy for the prevention and treatment of BPH is gaining popularity [11] due to its promising efficacy, milder side effects and affordability compared to most other treatment options. Anti-BPH properties of some plants, including saw palmetto, *Pygeum africanum, Secale cereale* and *Phellodendron amurense*, have been validated by several scientific investigations [12–14] and are widely used for the prevention and treatment of the disease.

*Annona muricata*, commonly known as soursop, belongs to the Annonaceae family. The plant is widely known for its anticancer properties [15]. Aside this popular medicinal use, a wide range of ethnomedicinal activities have also been attributed to different parts of the plant owing to some of its properties including anti-inflammatory, antiproliferative, hypoglycemic, sedative, smooth muscle relaxant and antispasmodic effects [16,17]. Some indigenous communities in Africa including Nigeria use *A. muricata* in their folk medicine. Leaf extract of the plant is used to alleviate difficulty associated with urination in certain communities in Eastern part of Nigeria. Although Asare *et al.* [18] reported that the aqueous extract of the plant leaf exhibited antiproliferative activity against BPH-1 cells, there is still paucity of information on the possible usefulness of this plant in the treatment of BPH.

It has been shown that plant-derived medications exhibit their anti-BPH effect through different mechanisms including antiandrogenic, antiproliferative, anti-inflammatory and antioxidant activities [8,19–21]. Antioxidants are known to mitigate the deleterious effect of oxidative stress - a factor believed to play a great role in the development of age-related diseases such as BPH [22,23]. In addition to antiproliferative effect, *in vitro* studies have indicated that *A. muricata* leaf extract also exhibits a remarkable antioxidant activity [24,25]. Thus, investigation of the effect of this plant material on BPH alongside its antioxidant activity could provide more useful information on its potential anti-BPH properties.

Annonaceous acetogenins are the major constituents of *A. muricata* and many pharmacological activities of the plant have been attributed to these bioactive constituents [15–17]. In this study, the effect of acetogenin-rich fraction of *A. muricata* leaves was investigated on testosterone propionate-induced BPH in Wistar rats through the measurement of antioxidant indices and some other biochemical parameters including prostate-specific antigen, prostate weight, relative prostate weight and prostate protein content.

## 2. Materials and methods

### 2.1. Chemicals and drug

The testosterone propionate (TP) used for the induction of BPH was purchased from Sigma-Aldrich chemical company, Germany. The enzyme-linked immunosorbent assay test kit used for PSA determination was a product of Elabscience Biotechnology, Inc. Houston, Texas, United State. Avodart^TM^ (Dutasteride) was manufactured by GlaxoSmithKline Pharmaceuticals S.A., 189 Grunwaldzka Str, 60–322 Poznan, Poland. Reagents used for the assays were commercial test kits purchased from Randox Laboratories Ltd., Crumlin, Antrim, UK. Thiobarbituric acid (TBA) was purchased from HiMedia Laboratories Pvt. Ltd, Mumbai, India. All other chemicals and reagents used for this study were of analytical grade.

### 2.2. Sample collection and extraction

Fresh green leaves of *A. muricata* were collected from Ikwo local government area, Ebonyi State, Nigeria. The plant leaves were authenticated at the Department of Agriculture, Alex Ekwueme Federal University Ndufu-Alike. They were subsequently washed with clean water and air-dried at room temperature under shade. The dried leaves were milled into fine powder using a milling machine. About 560 g of the ground material was soaked in 2.5 L of ethanol (95.5%) for 72 h. The mixture was then filtered with Whatman filter paper (grade 1) and concentrated at 50°C to about 50 mL.

### 2.3. Fractionation of the extract

The ethanol extract was first partitioned in n-hexane (100 mL ×3) using a separating funnel. The ethanol layer was then concentrated to obtain a solid crude extract. The crude extract was subjected to column chromatography (open column, silica gel 60), using the mixture of solvents: hexane/ethyl acetate (93/7 v/v), dichloromethane: ethanol (93/7, 80/20 and 50/50 v/v). Eluted fractions were collected with 100 mL beakers and were tested for annonaceous acetogenin using Kedde reagents. Formation of pink to red-purple color on addition of Kedde reagents indicated the presence of annonaceous acetogenin [26,27]. Fractions tested positive with Kedde reagents were combined and concentrated at 50°C. The extract was exposed in 100 ml beakers for five days to ensure total evaporation of organic solvent before being used for the experiment.

It has been shown from previous studies that acetogenin fraction obtained by column chromatography of ethanol extract of *A. muricata* leaves contains predominantly annonaceous acetogenins [28,29]; however, the presence of little amount of flavonoid has equally been detected in the fraction [28].

### 2.4. Acute toxicity test

The median lethal dose (LD_50_) of AFAL was determined according to the method of Lorke [30]. Thirteen BALB/c albino mice weighing 20–22 g were used for the LD_50_. They were purchased from the Veterinary Medicine Department, University of Nigeria, Nsukka. The mice were kept in standard cages at the average ambient temperature of 26 ± 1°C, 12 h light −12 h dark cycle and were fed commercial mice chow and tap water *ad libitum*. In stage one, the animals were placed in three different groups of three mice each and were administered graded oral doses of 10, 100 and 1000 mg/kg body weight respectively of AFAL dissolved in a mixture of dimethyl sulfoxide (DMSO) and H_2_O (in the ratio of 0.5: 9.5 v/v). Then in stage two, three mice were placed in three different groups and were orally administered 1600, 2900 and 5000 mg/kg body weight of the dissolved AFAL, while one mouse served as control. The mice were observed for toxicity signs and possible death in each group within 24 h of administration for the lethal dose determination. The LD_50_ of AFAL was obtained at 2900 mg/kg body weight which was considered safe and, based on this, doses of 100 and 200 mg/kg were selected for the experiment.

### 2.5. Experimental design

Twenty-five male Wistar rats weighing 110–120 g were purchased from the Veterinary Medicine Department, University of Nigeria, Nsukka. They were maintained under standard environmental conditions and were allowed free access to commercial grower’s feed and clean water *ad libitum.* The rats were acclimatized for one week before randomly distributed into five groups of five rats each.

The study groups and treatment protocol used in this experiment are shown in Table 1. BPH was induced in the rats by subcutaneous injection of testosterone propionate (3 mg/kg body weight, dissolved in olive oil) for 28 days [31]. Successful induction of BPH was ascertained by testing PSA level of the rats on the 29th day. Then oral administration of the acetogenin-rich fraction dissolved in DMSO/H_2_O (0.5: 9.5 v/v) was done for one week. On the last day of the experiment (day 36th), the animals were fasted, weighed using a sensitive balance and bled by cardiac puncture. Blood samples were collected in plain sample bottles and centrifuged (3000 g for 15 min) for the separation of serum. The prostates were excised, washed with cold saline solution, dried with adsorbent paper and weighed on sensitive balance. One prostate per group was selected and a section of the dorsolateral lobe was processed for histology. The remaining prostates were used for biochemical analyses. Relative prostate weight of the rats was calculated using the method of Kalu *et al.* [10]: Relative prostate weight = (Total prostate weight/Final body weight) × 1000

**Table 1.** Study groups and treatment protocol.

Group	Subcutaneous injection	Oral administration
Normal	Olive oil (1 ml/kg)	1 ml/kg DMSO/H_2_0 (0.5: 9.5 v/v)
TP	TP (3 mg/kg)	1 ml/kg DMSO/H_2_0 (0.5: 9.5 v/v)
TP + Dutasteride	TP (3 mg/kg)	Dutasteride (0.5 mg/70 kg)
TP + AFAL (100 mg/kg)	TP (3 mg/kg)	AFAL (100 mg/kg)
TP + AFAL (200 mg/kg)	TP (3 mg/kg)	AFAL (200 mg/kg)

All animals were fed rat chow and allowed free access to water throughout the period of the experiment. TP = testosterone propionate, AFAL = acetogenin-rich fraction of *A*. *muricata* leaves.

### 2.6. Biochemical analyses

The level of PSA in the rat sera was determined following the Elabscience enzyme-linked immunosorbent assay (ELISA) test kit procedure. The prostate protein content of the rats was estimated by the method of Tiez [32]. Lipid peroxidation was estimated by measuring thiobarbituric acid reactive substance expressed in terms of malondialdehyde (MDA) according to the method described by Wallin *et al.* [33]. The serum antioxidant activities: superoxide dismutase (SOD), glutathione peroxidase (GPx) and catalase (CAT) were assayed by the methods of Arthur and Boyne [34], Paglia and Valentine [35] and Sinha [36], respectively. Reduced glutathione (GSH) was evaluated by the method of Exner *et al.* [37].

### 2.7. Histopathologic examination

The dorsolateral lobes of the prostates were fixed in 10% phosphate formalin solution for 48 h. The prostate tissues were then dehydrated in grades of ethanol, cleaned in xylene and embedded in paraffin wax. Each block of the tissue was sectioned at 5 µm using rotary microtone and stained with hematoxylin and eosin (H and E). The sections were subsequently viewed and photomicrographs were taken to evaluate the structural changes.

### 2.8. Statistical analysis

Statistical analysis was performed using the Statistical Package for the Social Sciences software (SPSS Institute, Armonk, NY, U.S.A.) version 20. The results were expressed as mean ± standard error of mean (SEM). The level of homogeneity among the groups was evaluated using one-way analysis of variance followed by post-hoc multiple comparisons with the Duncan’s tests to detect the significant differences between the groups at *p* < 0.05.

## 3. Results

### 3.1. Effect of the acetogenin-rich fraction on PSA level of rats sera

**Figure 1.**
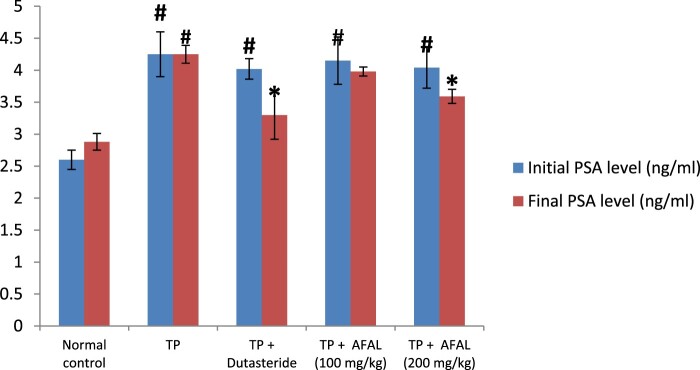
Effect of acetogenin-rich fraction of *Annona muricata* leaves (AFAL) on prostate-specific antigen (PSA) levels of testosterone propionate (TP)-induced BPH in rats. Values are expressed as mean ± standard error of mean (*n* = 5). ^**#**^Significant when compared to normal control (*p* < 0.05); *significant compared to TP control (*p* < 0.05).

Effect of the acetogenin-rich fraction of *A. muricata* leaves on PSA levels of the rats with experimentally induced BPH is shown in Figure 1. At the end of the 28 days of subcutaneous injection of testosterone propionate, there was a significant increase (*p* < 0.05) in PSA level (initial PSA) in groups that received the hormone compared to the normal control, indicating successful induction of BPH. Daily administration of the acetogenin fraction, at the dose level of 200 mg/kg body weight to the hyperplasic rats for seven days, caused a significant (*p* < 0.05) decrease in PSA level (final PSA) compared to the untreated rats (TP group). This result was not significantly different (*p* > 0.05) from those treated with the standard BPH drug (dutasteride). The ameliorating effect of the plant material on the induced BPH was in a dose-dependent manner.

### 3.2. Effect of the acetogenin-rich fraction on prostate and relative prostate weights of the rats

**Figure 2.**
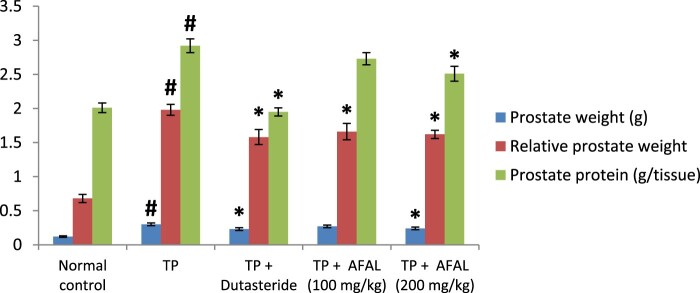
Effect of acetogenin-rich fraction of *Annona muricata* leaves (AFAL) on prostate weight, relative prostate weight and prostate protein content in testosterone propionate (TP)-induced BPH in rats. Values are expressed as mean ± standard error of mean (*n* = 5). **^#^**Significant when compared to normal control (*p* < 0.05); *significant compared to TP control (*p* < 0.05).

A significant increase (*p* < 0.05) in prostate weight and relative prostate weight was observed in the group that received the hormone alone when compared to the normal control, also validating that prostate hyperplasia was induced. A significant decrease (*p* < 0.05) in prostate weight, as well as relative prostate weight, was observed in the group treated with the acetogenin fraction when compared to the untreated rats (TP group). This indicated a remedial effect of the plant material on the induced BPH. Also, there was no significant difference (*p* > 0.05) in prostate and relative prostate weights between the test rats and those treated with the reference drug (TP + Dutasteride group) Figure 2.

### 3.3. Effect of the acetogenin-rich fraction on prostate protein content

The results showed that BPH induction significantly increased (*p* < 0.05) the prostate protein content as observed in the group that received the hormone alone, when compared to normal control. A significant decrease in prostate protein content was observed in the group treated with 200 mg/kg of the *A. muricata* acetogenin fraction when compared to the TP group. This value was not significantly different from the group treated with the standard drug. However, a non-significant decrease (*p* > 0.05) in the prostate protein content was observed in the group that received a lower concentration of the fraction (100 mg/kg) when compared to the TP group (Figure 2).

### 3.4. Effect of the acetogenin-rich fraction on oxidative stress parameters of the rat sera

**Figure 3.**
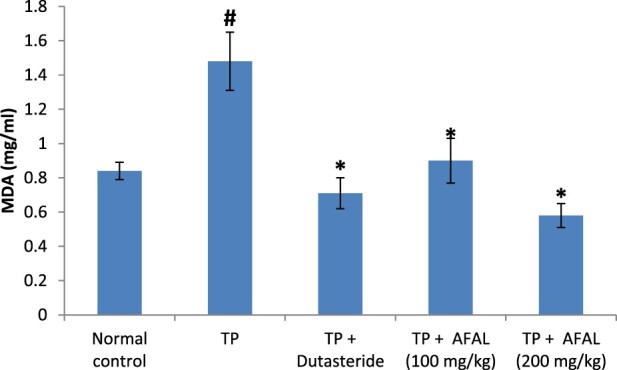
Effect of acetogenin-rich fraction of *Annona muricata* leaves (AFAL) on malondialdehyde (MDA) concentration in testosterone propionate (TP)-induced BPH in rats. Values are expressed as mean ± standard error of mean (*n* = 5). **^#^**Significant when compared to normal control (*p* < 0.05); *significant compared to TP control (*p* < 0.05).

**Figure 4.**
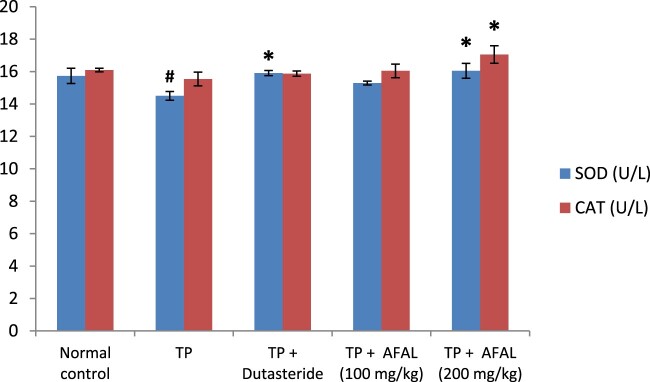
Effect of acetogenin-rich fraction of *Annona muricata* leaves (AFAL) on superoxide dismutase (SOD) and catalase (CAT) activities in testosterone propionate (TP)-induced BPH in rats. Values are expressed as mean ± standard error of mean (*n* = 5). **^#^**Significant when compared to normal control (*p* < 0.05); *significant compared to TP control (*p* < 0.05).

**Figure 5.**
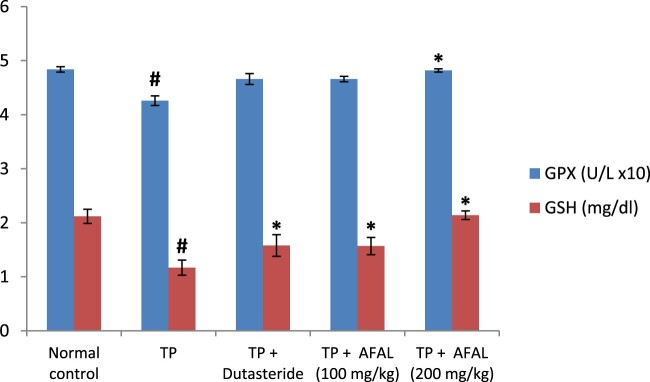
Effect of acetogenin-rich fraction of *Annona muricata* leaves (AFAL) on glutathione peroxidase (GPx) activity and reduced glutathione (GSH) concentration in testosterone propionate (TP)-induced BPH in rats. Values are expressed as mean ± standard error of mean (*n* = 5). **^#^**Significant when compared to normal control (*p* < 0.05); *significant compared to TP control (*p* < 0.05).

The effect of *A. muricata* acetogenin fraction on malondialdehyde (MDA) concentration and the activities of antioxidant enzymes were estimated as indices for oxidative stress status of the rats. Subcutaneous injection of testosterone propionate significantly increased the lipid peroxidation marker (MDA) of the group that received the hormone alone and as well decreased their antioxidant activities compared to the normal control group. Administration of the acetogenin fraction (200 mg/kg) reversed the impact of the exogenous hormone as the MDA concentration significantly reduced (Figure 3) and the antioxidant activities of the rat were elevated (Figures 4 and 5). The obtained values were comparable to the normal control and group administered the standard drug.

### 3.5. Histologic examination

**Figure 6.**
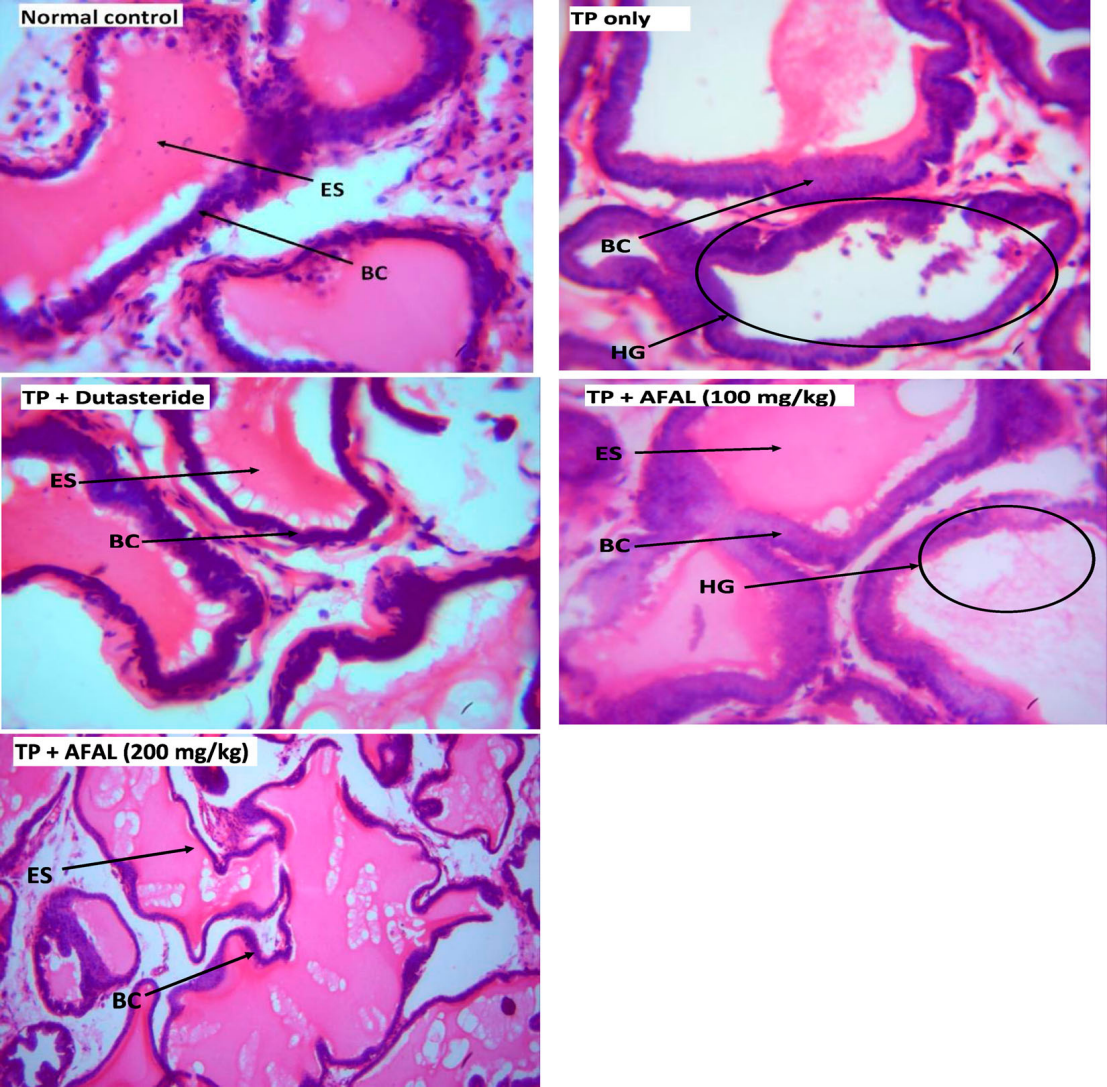
Micrograph of prostates in the experimental rats (Stain: H & E; Magnification: ×400). TP = testosterone propionate, AFAL = acetogenin-rich fraction of *A*. *muricata* leaves, ES = eosinophilic secretion, BC = basal cell and HG = hyperplastic gland.

The histologic section of prostate in normal group showed a standard prostatic architecture with prostate gland lined by basal cell (BC) and eosinophilic secretion (ES) within the alveoli. In the group that received the hormone alone, there was a severe hyperplastic gland (HG), change in morphology of the BC and secretion atrophy within the lumen. Treatment of the rats with 200 mg/kg *A. muricata* acetogenin fraction showed near-normal architecture with well-perfused secretion. This restoration is similar to the histoarchitecture of prostate in group treated with a standard drug, Figure 6.

## 4. Discussion

Medicinal plants remain one of the potent sources of human health due to the bioactive compounds that are responsible for their various pharmacological activities. *A. muricata* is a traditional medicinal plant that has been reported to possess acetogenins as major phytoconstituents [38]. These chemical compounds have been shown to exhibit various biological activities including the cytotoxic effect against the neoplastic cells which suggested their potential use as an antitumor agent [39].

In this study, BPH induction by subcutaneous administration of testosterone propionate (3 mg/kg) to the rats for 28 days was successful as evidenced in the rise of serum PSA level and prostate weights of rats that received the hormone alone. PSA is a glycoprotein that is predominately formed in the prostate gland and a reliable marker for BPH [40]. A significant reduction in the final PSA observed in test rats treated with the acetogenin-rich fraction of *A. muricata* (200 mg/kg body weight) is a pointer to the ameliorative effect of the plant material on the induced BPH. Decrease of PSA level is associated with a reduction of prostatic hyperplasia as a direct consequence of 5α-reductase inhibition [41]. Also, persisted high level of PSA (final PSA) in the group that received the hormone alone without any treatment is an indication that the observed drop in PSA level of the treated rats was not due to self-recovery. Comparing the level of decrease in the final PSA after the 7 days treatment suggests that AFAL (200 mg/kg body weight) exhibited a similar ameliorative effect as dutasteride on the induced BPH, possibly through 5α-reductase inhibitory activity among other mechanisms.

Furthermore, prostate enlargement is also an important marker for BPH [42]. The enlargement is due to the increase in both epithelial and stromal cell number (hyperplasia), resulting in an increase of prostate weight. Thus, the significant reduction of prostate weight of the BPH rat to near normal after treatment with the acetogenin-rich fraction (200 mg/kg) is also suggestive of a remedial effect of the plant material on the induced BPH. The results of the relative prostate weights are equally in agreement with the above observation; the relative prostate weight of rats that received hormone alone increased significantly in comparison with normal control, but treatment with the acetogenin fraction reduced the relative prostate weight to a near-normal weight.

BPH has been associated with increase in prostate protein content [43] and the observation of the present study was in accordance; rats that were injected with the hormone alone had elevated prostate protein content. Administration of the BPH rats with acetogenin fraction of *A. muricata* significantly decreased the protein content of their prostate which translates to attenuation of the induced hyperplasia.

The histologic findings from this study also affirmed a possible anti-BPH effect of the acetogenin-rich fraction of *A. muricata*, as prostatic histoarchitecture of the rats that received 200 mg/kg of AFAL showed recovery from prostatic hyperplasia when compared to the untreated rats.

Several literatures have associated oxidative stress with BPH development [23]; this could arise as a result of overproduction of oxidant molecules or depletion in the antioxidant system during prostate enlargement. Besides the possible prostate tissue damage by the reactive oxygen species, a compensatory cellular proliferation can as well set in, thereby worsening the prostate enlargement [23]. A balance between oxidative stress and antioxidant system of the cells, however, plays an important role in the development of prostate disease [44]. In this study, a significant elevation in the concentration of MDA with a corresponding decrease in the antioxidant activities observed in the rats that received the hormone alone confirmed associated oxidative stress in BPH development. This could be due to the impact of the supernormal dose of the exogenous hormone and physiological changes associated with prostate enlargement. However, administration of AFAL significantly decreased the MDA concentration and restored the antioxidant activities of the test rats in a dose-dependent manner.

Dutasteride is a known potent BPH drug whose mechanism of action is specific for the inhibition of 5α-reductase [45]. This was evident in this study where the drug obviously exhibited a higher ameliorative effect in BPH markers (PSA levels, prostate weight, relative prostate weight and prostate protein content) compared to AFAL, though the differences were not statistically significant. However, superior antioxidant capacity of rats treated with AFAL (200 mg/kg dose) compared to the standard drug is notable. This suggests that reinforcement of the antioxidant system could be one of the possible mechanisms through which the plant material was able to attenuate the induced BPH. Further studies, however, are recommended to elucidate other possible mechanisms of action of this plant material on BPH.

## 5. Conclusion

The results of this study confirmed that acetogenin-rich fraction isolated from *Annona muricata* leaves could alleviate BPH induced in Wistar rats. At the dose of 200 mg/kg, the extract clearly reversed the effect of the supernormal dose of testosterone propionate on the Wistar rats. Enhanced antioxidant system may be one of the mechanisms through which the plant material was able to exert its effect on the induced BPH. Therefore, acetogenin-rich fraction from *Annona muricata* leaves may be useful in the management of BPH.

## 6. Ethics approval

The laboratory animal protocol used in this study was approved by the Department of Biochemistry, University of Nigeria, Nsukka as well as the Faculty of Biological Sciences Ethics and Biosafety Committee of the same institution. The procedures agree with the guideline by the National Institutes of Health (NIH) publication on Guide for the Care and Use of Laboratory Animals.
